# Advancing health through evidence assisted decisions with health policy and systems research program: a qualitative evaluation of a national health research grant management process in the Philippines

**DOI:** 10.1186/s12961-023-01021-6

**Published:** 2023-07-13

**Authors:** Reneepearl Kim Sales, Gladys Kaye Reyes-Ramos, Chiqui de Veyra, Gabrielle Gascon, Vianka Barraca, Gillian Garcia, Maria Eufemia Yap

**Affiliations:** Alliance for Improving Health Outcomes, Veria 1 Building, 62 West Avenue, West Triangle, 1104 Quezon City, Philippines

**Keywords:** Research grants, Grant management, Health policy and systems research, Philippines, Research governance, Evaluation

## Abstract

**Background:**

Health policy and systems research (HPSR) has influenced Philippine policies, including tobacco control, mental health, and COVID-19. The Department of Health (DOH) Philippines and Philippine Council for Health Research and Development (PCHRD) launched the Advancing Health through Evidence-Assisted Decisions (AHEAD) with HPSR program in 2017, aiming to build a community of researchers and decision-makers committed to evidence production and utilization. Research systems employ grant management processes for transparency and accountability in research funding, preventing waste, fraud, and misuse of funds.

**Methods:**

This study evaluated AHEAD-HPSR's grant management using surveys, interviews, and focus groups to document (1) grant administration processes implemented by DOH and PCHRD, and (2) experiences of grantees, program managers, staff, and policymakers. Data were initially analyzed through the USA Grant Accountability Office’s Federal Grant Life Cycle, with new themes created as they emerged. The study identified processes and gaps in the research grant life cycle stages: design/redesign, pre-award, award, implementation, closeout, and research dissemination and utilization.

**Results:**

Identification of research areas for the grant are identified using national and departmental research priorities. While Calls for Proposals are posted publicly, researchers that have previously worked with policymakers are contacted directly to submit proposals. The evaluation found that research is delayed by bureaucracies in grant administration, particularly in financial reporting and ethics review processes. Complying with the terminal financial report was identified as the most challenging part of the grant process due to immense auditing requirements. Grantees recommend the simplification of bureaucracy for fund release to enable them to focus on research work.

**Conclusion:**

This study contributes to the limited literature on health research grant management in developing countries. Valuable information and recommendations were contributed by stakeholders in this evaluation. These are manifestations of a continuing interest and desire to make health policy and systems research in the Philippines more robust and relevant. It is imperative for the program to continually evolve and build systems most applicable to its multidisciplinary context.

## Introduction

Health systems are under increasing pressure to implement interventions that will improve population health. The 58th World Health Assembly in 2004 and the 2008 Bamaka Call to Action emphasized the use of evidence for policy as an essential priority for governments to achieve health equity [1, 2]. Evidence-based policymaking can increase the effectiveness of an intervention, lead to better resource efficiency, and manage expectations of what an intervention can do [3–5].

Health policy and systems research (HPSR) plays a pivotal role in shaping health systems and seeks to guide policies to achieve health goals [6]. The attainment of high quality research begins with a strong national health research system that generates, disseminates, and uses evidence [7]. In 2003, WHO developed a conceptual framework for health research systems that identifies its functions and operational components [8]: (1) stewardship, (2) financing, (3) creating and sustaining resources, and (4) producing and using research. A 2020 WHO Health Evidence Network Synthesis Report found that one of the key challenges in strengthening health research systems is the efficient use of available research resources for optimal benefit and impact [9, 10]. However, low- and middle-income countries (LMICs) also continue to have limited research resources. The 2017 World Report on HPSR revealed that funding and trained human resources were the most serious constraints to HPSR generation in LMICs [6]. As little as 10% of research institutions in LMICs have unrestricted, long term funding essential for sustainability of HPSR [11]. A 2021 report by the Alliance for HPSR found that HPSR budget is often unspecified in national health research funds, with a low share of domestic funding [12]. Where the research community is small with limited funding, such as in LMICs, there is a growing demand for efficiency in research and its use in policy and practice [13, 14]. Stewardship and governance in health research systems can aid accountable resource allocation and use through vision-setting, priority-setting, and system monitoring and evaluation [8]. This is crucial in LMICs, where external stakeholders and funders play a significant role in HPSR development [9, 14, 15].

In the Philippines, HPSR has shaped national policies, most recently in tobacco control, increasing taxation of alcohol products, mental health, and COVID-19 [16–19]. HPSR training is limited in frequency in the country [17]. The Department of Health (DOH) and Philippine Council for Health Research and Development (PCHRD) launched the Advancing Health through Evidence-Assisted Decisions with Health Policy and Systems Research (AHEAD-HPSR) program in 2017. The program envisions a community of researchers and decision makers committed to the production and use of evidence with the goals of [20]:

1. Informing the health sector's global and national administrative and legislative agenda;
2. Creating an equitable and enabling environment for HPSR, and;
3. Ensuring the progressive realization of the envisioned HPSR ecosystem.

The conduct of research is an identified component to achieve AHEAD-HPSR’s goal and is implemented through the grantmaking role of DOH and PCHRD [21]. Since 2017, the program has awarded 51 non-institutional grants (short-term funding to conduct 1 research) and 5 institutional grants (long-term funding to research institutions). The program has awarded an average of Php 3.6 million (approximately USD 66,000) per grant. Research conducted under the program have included national to local level studies using qualitative and quantitative methodologies.

Awards and grants contribute to a country’s research competitiveness. The USA National Institutes of Health (NIH) allotted over USD 24 billion (almost 59% of their budget) to research project grants alone in 2021. This was used to award 39,897 research project grants in 2021 (with an average size of USD 581,293) and represented 79% of all grants awarded by the USA NIH that year [22]. In 2021, UK Research and Innovation committed GBP 2.8 billion to research awards, resulting in over 4000 grants awarded [23]. For oversight of such vast amounts of resources, health research systems employ grant management processes that support governance by ensuring transparency and accountability in the research funding process. This can include government-wide policies and regulations related to grant application, awarding, and allowable research spending, among others [9, 24]. Grantees also have to comply with various reporting requirements throughout the duration of their grant. These requirements and policies work towards preventing research waste, fraud, and misuse of funds [24].

Although accountability policies in research grants are necessary, compliance can result in significant burden to researchers. Interviews with administrative staff, researchers, and stakeholder organizations in the USA revealed 3 factors that contribute to the burden of compliance with grant policies: (1) changing implementation of requirements, (2) detailed requirements to develop and submit documents for proposals, and (3) increased prescriptiveness of requirements. These factors resulted in increasing workload and costs to researchers by (1) having to hire administrative staff that specialize in funder policies to ensure compliance and (2) researchers spending time to study requirements and reformat applications or reports [24]. A 2012 survey of grant recipients reported that 42% of their time on average is spent meeting grant requirements rather than actual research work [25]. The 2022 independent review of research bureaucracy in the UK revealed non-essential bureaucracy in overcomplicated requirements, unnecessary approval hierarchies in universities due to risk aversion, and poor communication of rationale for requirements to grantees. The report reiterated that non-essential bureaucracy should be eliminated to limit impediments in publicly funded research, thus avoiding wastage of funds [26].

From experiences in the USA and UK, high income countries with large research resources also struggle with efficient grant management. Given the important role of grant management in the stewardship of health research systems, this study aimed to evaluate the research grant management of the AHEAD-HPSR program. The evaluation can inform future efforts to optimize research grant management in LMICs towards supporting the generation of evidence-based health policies.

### The AHEAD-HPSR grant management process

**Fig. 1:**
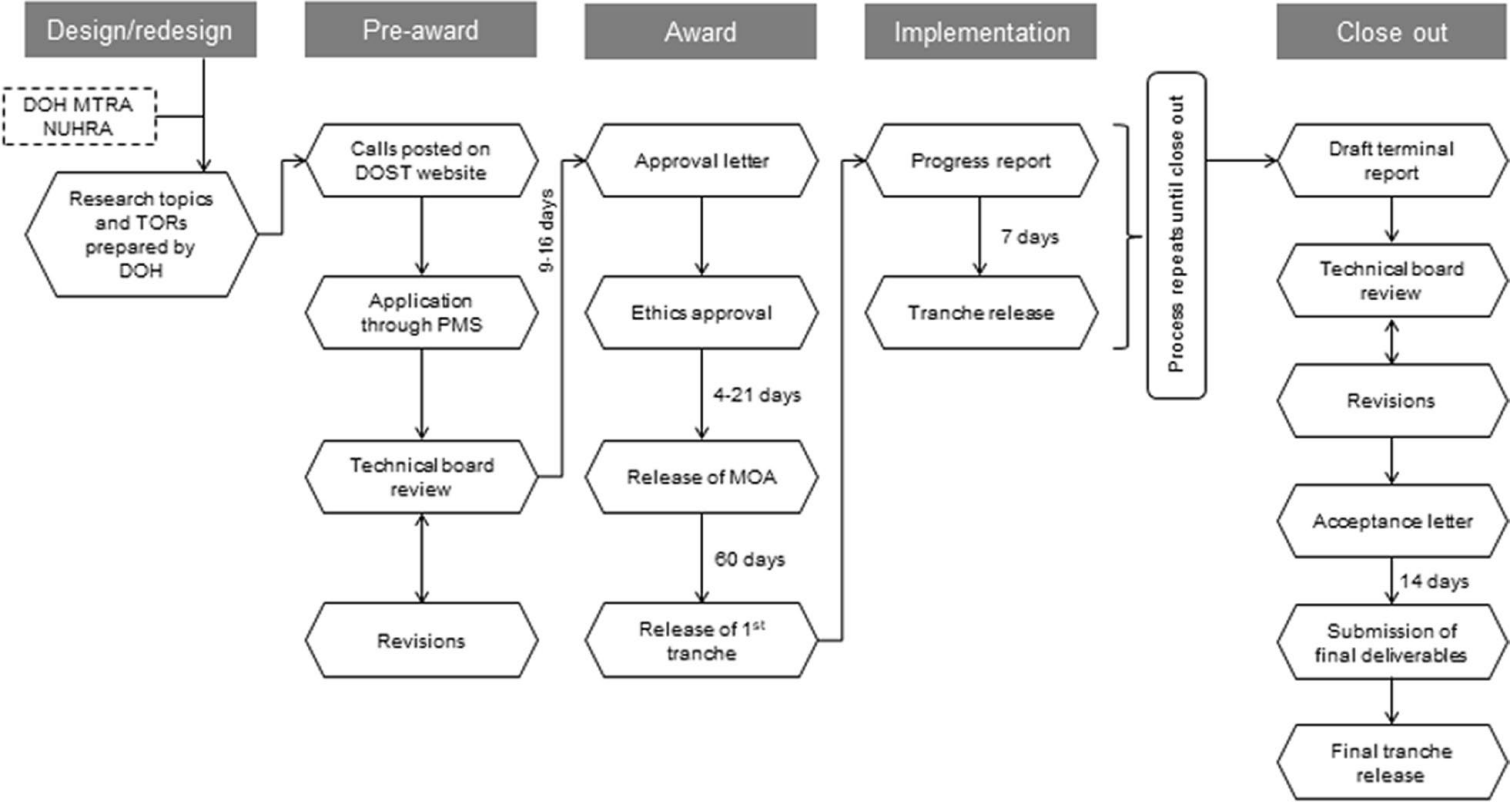
AHEAD-HPSR grant management process

Prior to the evaluation, the AHEAD-HPSR grant program was situated within a normative framework of a grant management process using the 2006 and 2016 versions of the United States Grant Accountability Office’s Federal Grant Life Cycle [27, 28]. The grant management process of the AHEAD-HPSR program occurs in five stages (Fig. 1): (1) Design/redesign, (2) Pre-award, (3) Award, (4) Implementation, and (5) Close out. The research topics under the program must be drafted to align with the DOH Medium Term Research Agenda (MTRA) and the current version of the National Unified Health Research Agenda (NUHRA) [20]. Terms of References (TOR) are drafted by DOH and these are posted in the PCHRD website and their regional counterparts [29, 30]. As part of the application, applicants must submit (1) a letter of intent, (2) proposal, (3) duties and responsibilities of project personnel, and (4) institutional profile. The proposals are reviewed by a technical board managed by DOH and PCHRD based on the following criteria: (1) technical merit, (2) data management, (3) significance, (4) feasibility, and (5) proponent or institutional capacity [21]. The selected applicant receives an approval letter and the Memorandum of Agreement (MOA) and ethics clearance must be accomplished before the first tranche of funding is received [21]. Throughout implementation, a quarterly progress report is submitted to PCHRD and DOH, and a meeting is held between all parties to discuss implementation progress before the subsequent fund tranches can be released. At the close out stage, the draft terminal report is reviewed by a technical review board [20]. Once revisions have been accepted, final versions of all deliverables must be submitted by the proponent. The final fund tranche will be released once these are received by PCHRD [21].

## Methods

This study evaluated the research grant management of the AHEAD-HPSR program with the aim of documenting (1) its grant administration process and (2) the experiences of grantees, grant program managers, grant program staff, and DOH bureaus/policymakers with the AHEAD-HPSR grant program. Data was collected through surveys, key informant interviews (KII), and focus group discussions (FGD).

### Participants

Participants were selected using purposive and convenience sampling through the details provided by the AHEAD-HPSR program. Grantees were purposively sampled based on their type of organization, grant amount received, and grant duration to ensure variety among participants. Snowball sampling emerged during data collection as respondents recommended other relevant individuals to participate in the study. Participants were contacted via official email on 23 July 2020. This email contained information on the evaluation purpose and methodology, proposed interview date, and their options for participation (survey, KII, or FGD). A follow-up email was sent after seven days if no response was received.

### Data collection

A topic guide was developed based on the indicators and metrics identified in the Grant Life Cycle for Federal Grant-Making Agencies and Grant Recipients through the United States Government Accountability Office and the National Health and Medical Research Council of the Australian government [28, 31]. Four different sets were prepared according to the type of respondent: (1) grantee, (2) AHEAD management, (3) AHEAD program staff, and (4) policymakers. The topic guide was pilot-tested three times then revised before data collection.

Data collection and analysis was conducted by 6 researchers (qualifications summarized in Table 1). Data was collected from 27 July to 24 September 2020. Due to movement and assembly restrictions during the COVID-19 pandemic, this study used online platforms and tools for data collection. Online surveys were deployed using Google Forms, while Google Meet was used for KIIs and FGD. Consent was sought from participants prior to the start of the KII or FGD. The KIIs and FGD lasted between 40 to 120 min and were audio, video, and chat recorded. A mix of English and Tagalog was used by interviewers to ask questions and by participants to respond. Only the participants and researchers were present in the call. No repeat interviews were conducted.

**Table 1:** Qualifications of researchers

Interviewer	Credentials and experience	Sex
RKS	More than 5 years experience in qualitative research, and research development and management Continuing education courses on Qualitative Research Methods Master of Science in International Public Health (included modules in Qualitative Research Methods)	Female
CDV	More than 5 years experience in quantitative and qualitative research, and research development and management Completed training courses on qualitative research approaches Bachelor of Science in Public Health	Female
GKR	Experience in qualitative research, including qualitative data collection Doctor of Medicine, including lectures on qualitative research methods Masters of Business Administration Bachelor of Arts in Independent Studies with a focus on Global Health and International Development	Female
GG	Bachelor of Arts in Political Science	Female
GG	Experience in qualitative research and data collection in the fields of anthropology and public health Master of Arts in Archaeology Bachelor of Arts in Behavioral Sciences	Female
VB	Experience in qualitative research, specifically in conducting key informant interviews, focus group discussions. Also has experience in quantitative analysis Masters of Science in Clinical Psychology student Bachelor of Arts in Psychology	Female

### Data processing and analysis

Transcription was aided by Tactiq, a real-time transcription app with integration on Google Meet [32]. Each transcript generated by Tactiq was reviewed for accuracy and revised as necessary by 2 researchers using the recording within seven days after the KIIs or FGD. Survey responses were automatically generated by Google Forms into a Google Sheets format.

Qualitative data was managed through Dedoose, a qualitative data management software [33]. Data from the KIIs and FGD were analyzed using thematic and content analysis, guided by the method recommended by Creswell [34]. Thematic and content analysis use a systematic process of coding, examination of meaning, and provision of a description of the social reality through the creation of themes. Through an abductive approach, the themes and codes were categorized into the appropriate grant life cycle step. New themes were generated as they emerged.

Two researchers coded each transcript and a third coder was assigned to resolve any disagreements between the original two coders. The finalized codes were analyzed, collated, and organized into themes. Interpretation and conclusions from the data were developed with consideration for its context and relationships between themes.

The language in the transcript reflected the original language used in the interviews, which is a mix of English and Tagalog. However, in consideration of the global audience of this publication, the quotes in the results section are presented fully in English.

## Results

The study included 35 participants (Table 2). This included 11 non-governmental organizations (61%) and 7 academic institutions (39%). The study also included participants affiliated with government agencies, including 1 from the AHEAD-HPSR management team, 3 from the AHEAD-HPSR program staff, and 6 from DOH bureaus.

**Table 2:** Participant characteristics

Type of participant	No. of participants and type of participation	Type of institution	Type of grant
Grantee	KII: 11 (61%) Survey: 7 (39%)	Non-governmental organization: 11 (61%) Academe: 7 (39%)	Institutional: 5 (28%) Non-institutional: 13 (72%)
AHEAD-HPSR management	KII: 1 (25%) Survey: 3 (75%)	Government: 4 (100%)	N/A
AHEAD-HPSR program staff	FGD: 4 (31%) KII: 3 (23%) Survey: 6 (46%)	Government: 11 (85%) Former staff: 2 (15%)	N/A
DOH bureau	FGD: 6 (100%)	Government: 6 (100%)	N/A

Table 3 presents a summary of key findings per grant life cycle step and recommendations. The following section details the results of the evaluation.

**Table 3:** Summary of key findings per grant life cycle step and recommendations

Grant life cycle step	Key findings
Design/redesign	• The identification of research topics for the AHEAD-HPSR program is mainly based on the DOH MTRA and NUHRA, with additional consideration given to the DOH Integrated Health Agenda, health priorities set by the National Objectives of Health or Secretary of Health, and urgent concerns from the administration due to public health emergencies • The different DOH bureaus, as end users of research, conduct their own prioritization process, and HPDPB compiles the priorities and needs of each bureau to produce a shortlist of topics to be presented to PCHRD • The TOR endorsement process takes an average of six weeks, but can be delayed by more than two months due to factors such as availability, responsiveness, and changes in leadership or grant management
Pre-award	• Calls for Proposal are announced online through the PCHRD website and email blasts to research institutions. The standard information included in the Call are the TOR, application forms, and budget ceiling • Researchers with a working relationship with either PCHRD or DOH receive Calls for Proposals through email or are contacted directly to submit a proposal. First time applicants rely on announcements through other channels to begin proposal development • The selection of grantees is based on a system using an evaluation form or scorecard that assesses the significance, relevance, and technical soundness of the proposal • Grantees noted that technical review board comments tend to focus on the budget instead of the methodological aspects of the proposal. Reviewer availability and responsiveness were identified as barriers in the timely release of technical clearance
Award	• PCHRD communicates grant decisions to applicants formally through an approval or rejection letter, and informally via email. Program staff reported that legal clearance causes delays in MOA preparation, which results in a longer waiting time for grantees to receive the final MOA • Most grantees complain about having to go through the process of two review boards: technical and ethics. The ethics review process is slow and causes delays to project implementation, and grantees reported minimal improvement in ethics clearance turnaround time even with new guidelines • Grantees also noted an unnecessary overlap in the review process, since ethics boards also comment on technical aspects of the proposal which were supposedly under the purview of the technical review panel
Implementation	• Familiarity or previous experience in being a grant recipient allowed grantees to directly contact AHEAD-HPSR managers for questions and grant issues without going through their assigned project officers • Delays in receiving funds are caused by 1) errors in entry of forms, financial reports, and the line item budget, 2) delayed ethics approval, 3) bureaucratic institutional processes, and 4) meticulous scrutiny of reimbursement receipts • Changes in scope of work such as expanded deliverables not included in the approved proposal, grant management policy changes during implementation, and other varying ethical requirements also hinder time-bound research implementation
Closeout	• Grantees find the completion of the terminal financial report as the most challenging part of the grant process due to auditing requirements • In rare cases of failure to submit financial reports and other requirements, the project could be suspended, terminated, and have its funding discontinued
Research dissemination and utilization	• The research dissemination and utilization activities occur after the submission and approval of the final report. Funding is available for publication and conference presentations through PCHRD • Dissemination materials are shared with relevant DOH bureaus to support health policy and program development, and utilization is monitored by both PCHRD and DOH
Recommendations	• Informants recommended seminars on grant policies to ease the bureaucratic burden of grant management • Simplifying the bureaucracy and the release of funds is necessary to enable researchers to focus on their work • Both technical and ethical reviews can be accelerated through the hiring of in-house reviewers, and agreements with review boards should be initiated to expedite these processes • Improving research dissemination is a key recommendation. The grant program can publish its own journal to increase publication from its grantees, simplify the information produced by research, and cultivate a research culture among policymakers that will encourage them to practice evidence-based policymaking

### Design/redesign

Identification of research topics for the program are based mainly on the DOH MTRA and NUHRA. The DOH Integrated Health Agenda, health priorities set by the National Objectives of Health or Secretary of Health, and other urgent concerns from the administration as a result of public health emergencies are also considered.

“Actually, both the Health Policy and Development and Planning Bureau (HPDPB) and PCHRD are also members of the group on Science and Technology of the National Task Force, and one of our critical tasks is the consolidation of research agenda of different agencies that involve research and development related to COVID. So there are research priority areas that are identified by PCHRD, then there are also research priority areas identified by DOH. All of us are informed about that type of situation and we’re very flexible, especially when there’s urgency and need to do those types of projects.”—AHEAD Management

“We make sure that the priorities…all of the grants we give out are aligned with the NUHRA. Then we also do our own in-house evaluation. We have our own template for the evaluation, and then after the evaluation, we get clearance from our immediate supervisor or Division Chief. Then our division chief will endorse it to the Executive Director for approval.”—AHEAD Staff

The different DOH bureaus, as end users of research, are expected to conduct their own prioritization process. HPDPB compiles the priorities and needs of each bureau and produces a shortlist of topics that will be presented to PCHRD. Consultations with different bureaus are held quarterly to check if the research priorities have changed before initiating another call.

“It’s (the Terms of References) basically decided by HPDPB but they usually talk to the different bureaus, different divisions. I think they even talk to the (DOH offices) in the region, requesting for their programs, research they want to do, and issues they need to address. They request all of the bureaus and divisions to forward a list to HPDPB, which the HPDPB will assess and merge similar topics before finalizing. So they (DOH) also make the TOR, but then the TOR signatories are both the HPDPB and PCHRD.”—AHEAD Management

“We also get suggestions from the (DOH) offices. I guess for the research they want to be done. And we make sure it’s aligned to our MTRA. Because the MTRA is basically…it’s not very specific, it’s more general areas. So there’s a large space for (DOH) offices to see what their needs are based on the agenda indicated in the MTRA.”—AHEAD Staff

Terms of References (TOR) development is led by HPDPB, in coordination with the different bureaus. PCHRD provides assistance by determining the feasibility of the research based on available funds. The DOH bureau is responsible for formally endorsing TORs for inclusion in the current fiscal year. This process takes, on average, six weeks. However, factors like availability, responsiveness, and change in leadership and management from the end of the DOH bureaus can delay the process by more than two months.

### Pre-award

Calls for Proposal are announced online through the PCHRD website and email blasts to research institutions. Standard information included in the Call are the TOR, application forms, and budget ceiling. TORs or Calls are reposted if they receive no submissions by the indicated deadline. Alternatively, these are forwarded to identified experts to directly request a proposal submission.

“There are (DOH) offices who know someone that can take on a project or they have worked with before. But even so, it will still undergo the screening process.”—AHEAD Staff

Researchers with a working relationship with either PCHRD or HPDPB receive Calls for Proposals through email or were contacted directly to submit a proposal. This is in contrast to first time applicants who rely on announcements through other channels to begin proposal development.

“Well we heard it from the DOH. We had a project with them and then we were informed that there's this AHEAD for HPSR Call for Proposals. And so, well, we responded to it and we prepared and submitted the proposal.”—Grantee

Proposal submissions are received through PCHRD’s online project management system. A technical review board composed of at least 3 members assesses the proposals. The relevant DOH bureau automatically fills one slot of the review board and experts from the public or private sector are selected from an existing pool by PCHRD. International reviewers may be invited, but this is rare and has only happened once.

The selection of grantees is based on a ranking system using an evaluation form or scorecard that assesses the significance, relevance, and technical soundness of the proposal. The contribution of the proposed study to the program, applicant’s track-record, and support from relevant DOH bureau/s are other considerations. The relevant DOH bureau/s serves as the final decision maker in grantee selection.

Technical review board comments are forwarded to the applicants. Information from grantees reveal that the comments tend to focus on the budget instead of methodological aspects of the proposal. While turnaround time of revisions by grantees are strictly monitored, the same cannot be said for the technical review board comments. Reviewer availability and responsiveness were identified as barriers in the timely release of technical clearance. As such, program officers prefer the organization of an en banc meeting, wherein the applicant and all reviewers are present to immediately give feedback on the proposal.

“So proponents are guided or whoever submitted the proposal. They present in an en banc meeting. They are physically there and they discuss their proposal. So brainstorming can happen there and (DOH) offices are also invited.”—AHEAD Staff

### Award

PCHRD communicates decisions to applicants formally through an approval or rejection letter and informally via email. MOA development is carried out by PCHRD project officers. Program staff reported that this process takes less than a month but grantees report a waiting time of two months to receive the final MOA. Legal clearance causes the delay in MOA preparation, according to program staff.

Grantees are required to undergo ethics review once the proposal has been approved by the technical review board and before signing the MOA. Although the ethics review process is done simultaneously with the application for the Science Foundation Unit Certification and MOA development, most grantees complain about having to go through the process of two review boards: technical and ethics. The ethics review process is slow and causes delays to project implementation.

“Well, I think that's where we really have a gap—that it's very easy to call for proposals and ask for proposals. But the real challenge is actually getting the technical and ethical review expedited. I just found the technical and ethics review difficult because we are really at the mercy of the availability of reviewers.”—Grantee

Grantees reported minimal improvement in ethics clearance turnaround time even with new guidelines, primarily because review is still dependent on ethics board speed and availability. Grantees noticed an unnecessary overlap in the review process, since ethics boards also comment on technical aspects of the proposal, which were supposedly under the purview of the technical review board.

### Implementation

Grantees receive supervision from their assigned project officer throughout research implementation. Frequency of communication varies from weekly to a few times per month via email, phone call, or text messages. Familiarity or previous experience in being a grant recipient also allowed grantees to directly contact AHEAD-HPSR managers for questions and grant issues without going through their assigned project officers. Such familiarity provides faster responses and bypasses any unnecessary communication that delays research implementation.

Grantees are required to submit quarterly technical and financial reports but the timeline for feedback received on these reports are lengthy. Staff turnovers are common in AHEAD and a lack of a proper staff turnover process was lamented by both AHEAD staff and grantees. Projects handled by the outgoing staff were only endorsed through soft and hard copies of documents, along with the latest updates regarding the project. This parallels grantee experience of not knowing they were reassigned to a new project officer, who had to be reoriented by grantees on their research.

“The turnover is literally just passing over of files. The electronic copy, hardcopies, and the latest updates. For example, project A, this is the update. Project B, this is the update. But I wasn’t taught how to prepare this, how to prepare that, how and what the requirements are for this. I was just given the Grants-In-Aid Guidelines then it’s like ‘oh it’s up to you to read this’ or like ‘just read that’.”—AHEAD Staff

“There was a problem when they changed the project manager. You need to orient the person again. They need to get used to your project…understand the project. —Grantee

PCHRD releases the project tranche after reports are submitted. However, some grantees experience delays in receiving funds due to (1) difficulty in collecting signatures, (2) delayed ethics approval, (3) bureaucratic institutional processes, and (4) meticulous scrutiny of reimbursement receipts. From the grant management perspective, such delays in fund release are caused by errors in entry of forms, financial reports, and the line item budget. There have also been instances when grantees experienced changes in their scope of work (such as expanded deliverables not included in the approved proposal), grant policy changes during implementation, and other varying ethical requirements depending on the research study sites. These are all lengthy processes that hinder time-bound research implementation.

“Midway through their project, the Department of Science and Technology secretary has required that new research proponents have to be foundations, have to be nonprofit. So I remember during the year 1 review, the secretary had questioned whether we’re profit or nonprofit. Good thing he was told we applied before the new guidelines were in place. We were given the job before the new guidelines were in place. But it's just an example of how the Department of Science and Technology can change the guidelines even if this is DOH money.”—Grantee

“It was postponed because of some policy changes in the funding institution, so that delayed the smooth-sailing part of phase 1. Changing some of the policies would be…I think will certainly affect and disrupt the implementation of the project. So I think that's one thing that should have been prevented. We should not change policies, right? Midway or during the course of implementation. That should be clear from the very beginning, that these are the (grant) policies.”—Grantee

### Closeout

The closeout process begins once the terminal technical and financial reports are submitted. Terminal reports are passed to HPDPB, the technical review board, and end users for review using the objectives indicated in the MOA. There is no existing scoresheet or rubric to check the quality of the terminal reports. Grantees find the completion of the terminal financial report as the most challenging part of the grant process due to auditing requirements. The turnaround time, including revisions, may take between 3 to 4 weeks. Clearance of final outputs are given by the end users, HPDPB, and the technical review board to achieve project completion. In rare cases of failure to submit financial reports and other requirements, the grantee project could be suspended, terminated, and have its funding discontinued.

“Usually, in PCHRD, we just look at the terminal report if you met your objectives, if your deliverables and target were as indicated in the MOA. We refer if any of these were not met. And then final reporting happens with a panel. Whoever reviewed the proposal will also be the panel that evaluates the final report because they already know about the proposal.”—AHEAD Staff

“I do understand that they have all these red tape to discourage corruption, but at the same time it also adds difficulties to the way researchers can do their work. We are not accountants. We're not finance people. We're researchers but we have to know a lot about finance. I think I wrote it in the form of AHEAD that they can also give us an orientation about the financial aspects of being a researcher for AHEAD. There's no such orientation, which is why we make mistakes in our reporting. It may be wrong, the way we're documenting things. Supporting documents may not be acceptable to the auditors, things like that.”—Grantee

### Research dissemination and utilization

During data analysis, it emerged that further activities occurred after the submission and approval of the final report. These activities relate to research communication, dissemination, and utilization. Respondents identified publications, forums, policy briefs, and infographics as usual formats of research dissemination. Although publication is not a requirement for all grantees, funding is available for publication fee and conference presentations through PCHRD. Dissemination materials are shared with the relevant DOH bureau/s to support health policy and program development. Utilization is monitored by both PCHRD and DOH, though not all outputs are immediately utilized. Improvement in tracking utilization was deemed necessary by HPDPB.

“Research dissemination and policy support are embedded. Meaning you produce policy briefs and policy statements and policy critique. And then research dissemination, you present, you share the findings to the public, whether in academic conferences and the public, etc. And then you produce. We need to produce, for example, academic, scientific publications and popular publications. For example, the manual that we developed, a popular publication, it's now being used by the rehabilitation and treatment centers. And then continue policy advocacy of course.”—Grantee

### Recommendations to improve the AHEAD-HPSR grant management process

Informants recommended seminars on grant policies to ease the bureaucratic burden on grantees, as well as the simplification of fund release processes.

“Simplify the bureaucracy so that the scientists can do their work. We have so few scientists and their hours are cut in half because now we need to do bureaucracy and accounting and micro accounting.”—Grantee

“If they can simplify the release of those funds. I know there are reasons they have all those bureaucracies—to remove corruption. But hopefully, for researchers they have to look at other models. Because in our country, to buy a computer takes two months. To buy reagents, another two months. There are so many obstacles.”—Grantee

Both technical and ethical reviews can be accelerated through the hiring of in-house reviewers. The program should have more authority and initiate agreements with review boards to expedite these processes.

“Hire good reviewers, hire good technical reviewers and they have to do it full-time.”—Grantee

Improving research dissemination is a key recommendation. The grant program can publish its own journal to increase publication from its grantees. AHEAD-HPSR should also simplify the information produced by research so the public can appreciate and understand its importance in improving quality of life. These activities can build a network of researchers, scientists, public health experts, and policymakers that can push for improving evidence-based policy in the country. Most importantly, the program should cultivate a research culture among policymakers that will encourage them to practice evidence-based policymaking.

“So we need our own journals, either produce our own journals…well we need to produce more researchers first, encourage more researchers. And then we produce our own journals. That's the direction that I see. We need to produce more.”—Grantee

## Discussion

Capacity for research stewardship and governance in LMICs, including evaluation of a health research grant management process, has received almost no analytical attention [35, 36]. This study contributes to the limited literature on HPSR grant management in LMICs. The results show positive features of the AHEAD-HPSR program’s grant giving arm, but also shed light on challenges that need to be addressed if its funded research aims to support health policy decisions.

The establishment of research governance systems stem from the need for accountability with increasingly publicly funded research, involvement of vulnerable populations, and exposure of fraud [24, 37]. Over regulation, bureaucracy, and red tape is well-documented in research, particularly in the fields of clinical and biomedical sciences [24, 26, 38–44]. These studies criticize bureaucracy for its rigidity, especially of the ethics review process, which impacts research efficiency and performance. The results of this evaluation showed that a similar barrier exists for HPSR in the Philippines despite streamlining of ethics guidelines. The ethics review process in the Philippines has been described as being designed for clinical research [45]. HPSR is a broad research field, and some of its activities are not comparable to clinical research. In this case, ethics review requirements may not all and always be relevant to HPSR [46]. Grantees also described the ethics review process as slow, the timeliness of which is at the mercy of reviewer availability that inevitably delays research implementation. Our findings are supported by a 2021 qualitative study on how ethics review board practices shape research in the Philippines [45]. Given the current state of the ethics process in the country, researchers will continue to focus on practical ways to obtain approval, downgrading it merely to a requirement to receive research funds.

AHEAD-HPSR grantees were also discouraged by immense financial reporting requirements, referring to it as red tape and micro accounting which consumes hours that could have been spent doing research. This reflects the negative characteristics of bureaucracy that have been described as slow moving, procedure-obsessive, and bloated which blocks rather than facilitates the day-to-today business of citizens [47]. Backchanneling became enabling practices with grantees, which favors those who already have the network and experience to move around the bureaucratic system. This familiarity eases the burden to the government agency in ensuring that grantee outputs comply with standards and procedures. Compliance reflects the nature of civil servants to be more concerned with adhering to processes, which are immediate, defined by rules, and more easily defensible [47]. From here, it could be said that such an environment does not foster innovation, flexibility, and agility, particularly with new and young researchers. These can be a deterrent among researchers to flourish and thrive overall.

The AHEAD-HPSR program expects that evidence generated from its grants, increasing research capacity, and publication of findings through conferences and peer-reviewed journals will influence policymaking [21]. The evaluation found that after closeout, most efforts are geared towards research dissemination and there is no exact guidance for utilization. This is also observed in other LMICs, which pay most attention to research production over communication [48, 49]. Strengthening evidence utilization is only possible if policy makers have access to evidence that they need and understand [50]. As such, providing evidence through publications or conferences is an insufficient means for research to reach policymakers [51]. Literature suggests implementing practical mechanisms to enhance researcher-policymaker interactions, fostering shared understanding of policy issues and collaborating on solutions [52, 53]. The AHEAD-HPSR program addresses the long known research-policy gap of failing to meet information needs of policymakers by institutionalizing best practice interventions that (1) define and use national and departmental research priorities, (2) involve policymakers in grant design, and (3) facilitate researcher-policymaker interaction through meetings. However, monitoring how these mechanisms exactly translate to evidence use in policymaking is lacking. Current reporting requirements for the AHEAD-HPSR grant are focused on meeting research objectives and submission of financial reports. But post-closeout, AHEAD-HPSR could benefit from enhanced monitoring of research policy impact to improve funding accountability and efficiency. Grant managers can draw on several existing frameworks to measure policy impact of AHEAD-HPSR funded research [54–57]. While each framework has its weaknesses, the grant program must also consider its own limitations in monitoring. It is recommended that the program initiate monitoring through data collection methods such as reviews, online surveys, or interviews over resource-, data-, and expertise-intensive methods like monetization models.

## Conclusion

This study contributes to the limited literature on health research grant management in LMICs. It evaluated a national health research grant program that aims to improve HPSR capacity and evidence-informed policymaking in the Philippines. Much effort has already been placed since the AHEAD-HPSR program was launched, and there is a continuing recognition of its potential contributions to developing the research culture in the Philippine health sector. Quality health research is taking center stage, further emphasized by the many unknowns and challenges that the COVID 19 pandemic has brought about. Valuable information and recommendations were contributed by various stakeholders in this evaluation. These are manifestations of a continuing interest and desire to make HPSR in the Philippines more robust and relevant. It is imperative for the program and the larger HPSR system in the country to continually evolve and build systems most applicable to its multidisciplinary context. It is important to ensure that the process doesn’t end at the submission of final reports but that findings are utilized by appropriate policymakers to inform health decisions.

## Study limitations

The AHEAD-HPSR program charter reviewed to describe the AHEAD-HPSR grant management process is a draft and may be subject to changes in the future. Any changes made on the program charter may create inconsistencies with the results gathered in this evaluation.

Movement and physical assembly restrictions during the COVID-19 pandemic may have limited data collection, in particular KIIs and FGDs. The online format may have influenced the respondents, particularly to those who were uncomfortable or unfamiliar with the technology. This may have limited the establishment of a good rapport and trust between the interviewer and participants, which in turn may have affected the depth of responses received.

## Data Availability

The datasets generated and analyzed for this study are available from the corresponding author on reasonable request.

## Abbreviations

AHEAD: Advancing Health through Evidence-Assisted Decisions
DOH: Department of Health
FGD: Focus group discussions
HPDPB: Health Policy Development and Planning Bureau
HPSR: Health policy and systems research
KII: Key informant interview
LMIC: Low- and middle- income countries
MOA: Memorandum of Agreement
MTRA: Medium Term Research Agenda
NIH: National Institutes of Health
NUHRA: National Unified Health Research Agenda
PCHRD: Philippine Council for Health Research and Development
TOR: Terms of reference
